# Occurrence of tick-borne pathogens in questing *Ixodes ricinus* ticks from Wester Ross, Northwest Scotland

**DOI:** 10.1186/s13071-021-04946-5

**Published:** 2021-08-26

**Authors:** Fanny Olsthoorn, Hein Sprong, Manoj Fonville, Mara Rocchi, Jolyon Medlock, Lucy Gilbert, Jaboury Ghazoul

**Affiliations:** 1 Institute of Terrestrial Ecosystems, Department of Environmental Systems Science, ETH Zürich, Universitätstrasse 16, 8092 Zürich, Switzerland; 2 Centre for Infectious Disease Control, National Institute for Public Health and the Environment, Antonie van Leeuwenhoeklaan 9, 3720 MA Bilthoven, The Netherlands; 3 Moredun Research Institute, Pentland Science Park, Bush Loan, Penicuik, EH26 0PZ UK; 4 Medical Entomology and Zoonoses Ecology Group, Emergency Response Department Science and Technology, Public Health England, Porton Down, Salisbury, SP4 0JG Wiltshire UK; 5 Institute of Biodiversity, Animal Health and Comparative Medicine, Graham Kerr Building, University of Glasgow, Glasgow, G12 8QQ UK

**Keywords:** Lyme borreliosis, Anaplasmosis, Hard tick-borne relapsing fever, Tick-borne diseases, One Health, Cross-sectional study

## Abstract

**Background:**

Lyme borreliosis and other tick-borne diseases emerge from increased interactions between humans, other animals, and infected ticks. The risk of acquiring a tick-borne infection varies across space and time, so knowledge of the occurrence and prevalence of pathogens in ticks can facilitate disease diagnosis in a specific area and the implementation of mitigation measures and awareness campaigns. Here we identify the occurrence and prevalence of several pathogens in *Ixodes ricinus* ticks in Wester Ross, Northwest Scotland, a region of high tourism and tick exposure, yet data-poor in terms of tick-borne pathogens.

**Methods:**

Questing *I. ricinus* nymphs (*n* = 2828) were collected from 26 sites in 2018 and 2019 and tested for the presence of tick-borne pathogens using PCR-based methods. Prevalence was compared with other regions of Scotland, England, Wales, and the Netherlands.

**Results:**

*Anaplasma phagocytophilum* (4.7% prevalence), *Borrelia burgdorferi* sensu lato (s.l.) (2.2%), *Babesia* from clade X (0.2%), *Rickettsia helvetica* (0.04%), and *Spiroplasma ixodetis* (0.4%) were detected, but no *Neoehrlichia mikurensis*, *Borrelia miyamotoi*, or *Babesia microti*. Typing of *A. phagocytophilum* using a fragment of the *GroEL* gene identified the presence of both ecotype I and ecotype II. Genospecies identification of *Borrelia burgdorferi* s.l. revealed *B. afzelii* (53% of infected nymphs), *B. garinii* (9%), *B. burgdorferi* sensu stricto (7%), and *B. valaisiana* (31%). We found similar prevalence of *A. phagocytophilum* in Wester Ross as in the Netherlands, but higher than in other parts of Great Britain. We found lower *B. burgdorferi* s.l. prevalence than in England or the Netherlands, and similar to some other Scottish studies. We found higher prevalence of *B. valaisiana* and lower prevalence of *B. garinii* than in other Scottish studies. We found *S. ixodetis* at much lower prevalence than in the Netherlands, and *R. helvetica* at much lower prevalence than in England and the Netherlands.

**Conclusions:**

As far as we know, this is the first description of *S. ixodetis* in Great Britain. The results are relevant for disease surveillance and management for public and veterinary health. The findings can also aid in designing targeted public health campaigns and in raising awareness among outdoor recreationists and professionals.

**Graphical abstract:**

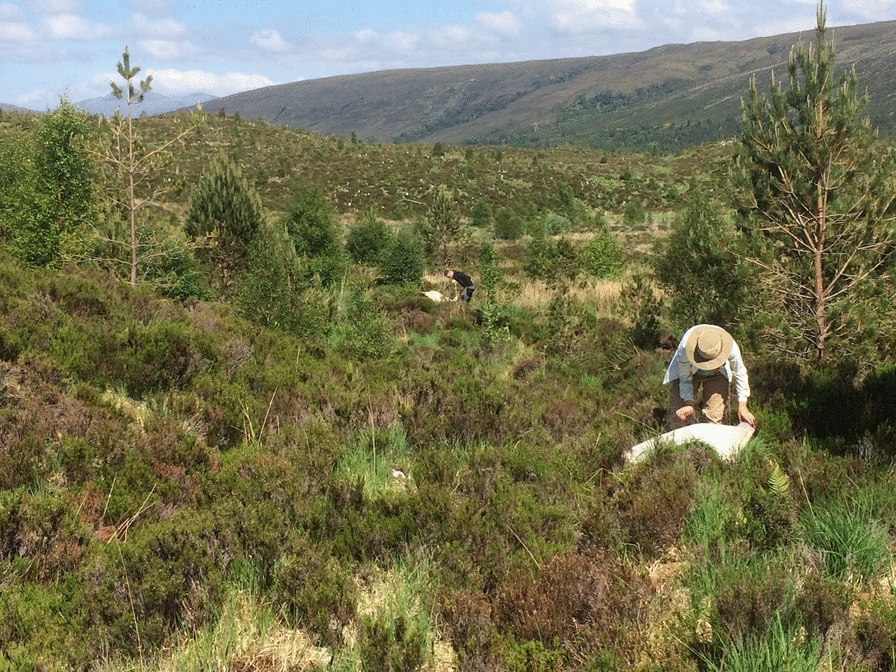

**Supplementary Information:**

The online version contains supplementary material available at 10.1186/s13071-021-04946-5.

## Background

*Ixodes ricinus* is the most abundant and widespread tick species in Europe [1] and, as a generalist feeder, transmits multiple pathogens of medical and veterinary importance [2]. The primary public health concerns are Lyme borreliosis, caused by *Borrelia burgdorferi* sensu lato (s.l.) infection, and tick-borne encephalitis (TBE), both of which have increased in incidence in several European countries in recent decades [3–7]. TBE virus was detected in the UK (England) for the first time in 2018 [8].

*Ixodes ricinus* also transmits other pathogens, including *Anaplasma phagocytophilum*, *Borrelia miyamotoi*, *Neoehrlichia mikurensis*, *Rickettsia helvetica*, *Spiroplasma ixodetis*, and several *Babesia* species. These are currently the key known pathogens that can cause disease in livestock, wild animals, and/or humans [9, 10]. Although the number of studies describing human infections involving these other tick-borne pathogens is increasing, their incidence is largely unknown, awareness is low, and adequate diagnostic modalities are often lacking in routine settings [10, 11].

Information on the geographical distribution and prevalence of pathogens in ticks helps to evaluate the risk of exposure through tick bites and, consequently, the risk of disease. This raises awareness of the appropriate tick-borne pathogens in areas of risk and can contribute to prevention, identification, diagnosis, and treatment of these diseases in humans and animals. This is particularly relevant to regions where the pathogens, and hence the accompanying diseases, are currently considered absent due to the lack of data on the presence and prevalence of these pathogens in ticks. For example, soon after becoming aware of the presence of *B. miyamotoi* and TBE virus in questing ticks in the Netherlands, local health professionals identified the first cases of hard tick-borne relapsing fever and TBE in human patients and could thus administer appropriate treatment [12, 13]. Awareness, accurate diagnosis, and hence treatment of a disease can therefore depend on the knowledge of the local presence of the pathogen in the environment.

One region in Europe that is data-deficient in terms of these emerging pathogens is Northwest Scotland. It is an area of potential high relevance to tick-borne pathogens, as it has high tick host populations, both wild, such as deer and small mammals, and livestock, especially sheep [14]. Northwest Scotland, including the Wester Ross area, is a sparsely populated region that has long been visited by tourists and is still growing in popularity. In 2019, 12 million visitors visited the Scottish Highlands [15], and Wester Ross is one of the most popular destinations in the Highlands, thanks to its dramatic mountain scenery and beaches. Many visitors engage in recreational outdoor activities that have potentially high exposure to ticks. Moreover, woodland expansion is being actively encouraged by government policies and supporting subsidies, and this is resulting in land cover changes [16] with potential consequences for the populations of ticks, their hosts, and hence the prevalence of tick-borne pathogens. We therefore consider Northwest Scotland to be of interest in view of the potential health concerns arising from disease caused by exposure to tick-borne pathogens.

The aim of this study is to provide a first assessment of the presence of a range of pathogens in questing *I. ricinus* ticks from Wester Ross, including a preliminary estimate of their prevalence. Specifically, we aim to test *I. ricinus* for the presence of *A. phagocytophilum, B. burgdorferi* s.l., *B. miyamotoi*, *N. mikurensis*, *R. helvetica*, *S. ixodetis*, *Ba. microti*, and *Babesia* species from clade X, formerly known as *Babesia* sensu stricto (s.s.) [17–19]. Samples positive for *A. phagocytophilum*, *B. burgdorferi* s.l., and *Babesia* species from clade X are further specified to the ecotype, genospecies, and species level, respectively. Importantly, we place these new presence and prevalence data in context by comparing them with previously published and other unpublished data from other regions of Scotland, England and Wales, and the Netherlands, as an example from continental Europe.

## Methods

### Tick collection

**Fig. 1:**
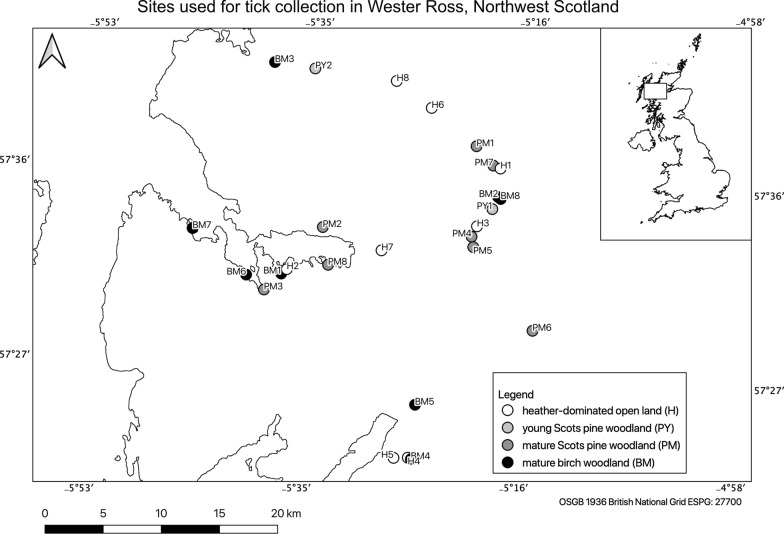
Geographical locations of the sites used for tick collection in Wester Ross, Northwest Scotland. The map was created using the free and open source QGIS 3.8. The location of the sites was recorded with a handheld GPS, the shapefile of the United Kingdom was obtained from Runfola D, Anderson A, Baier H, Crittenden M, Dowker E, Fuhrig S, et al. (2020) geoBoundaires: A global database of political administrative boundariies. PLoS ONE 15(4)

Ticks were collected from 26 sites in Wester Ross in Northwest Scotland, United Kingdom (57°24′–57°42′ N, 5°–5°54′ W), in 2018 and 2019 (Fig. 1). The four habitats of the sites were young Scots pine (*Pinus sylvestris*) woodlands (15–20 years old), mature Scots pine woodlands (minimum 50 years old), mature birch (*Betula* spp.) woodlands, and open moorland sites dominated by heather (*Calluna and Erica* spp.). A total of 2828 questing *I. ricinus* ticks (69 adult females, 72 adult males, and 2687 nymphs) were collected using the blanket drag/cloth lure method and kept for analysis in Eppendorf vials containing 70% ethanol. Previous studies have confirmed that 100% of the ticks identified from blanket drags in Scotland, including the Northwest region, are *I. ricinus*: in woodland sites across Scotland including Northwest Scotland, 2000 questing nymphs identified to species level were *I. ricinus* [20], in two studies carried out in the Northeast region of Scotland, 2500 and 1000 nymphs, respectively, identified to species level were *I. ricinus* [21], and in the North of Scotland, 4383 questing ticks (2700 larvae, 1550 nymphs, and 133 adults) identified were *I. ricinus* [22]. Therefore, we assumed that all ticks counted were *I. ricinus*. All the 2828 questing ticks were used for molecular analysis. For detailed information on the geographical location, land cover type, and times of data collection on the sites, see Fig. 1 and Additional file 1.

### Pathogen identification

Ticks were kept in Eppendorf vials filled with 70% ethanol until return from the field. All ticks (*n* = 2828) were then washed in distilled water, briefly dried, separated individually into polymerase chain reaction (PCR) plates, and kept at –20 °C until DNA extraction. All ticks were extracted with ammonium hydroxide as described previously [23]. The ticks were analysed individually for the presence of tick-borne pathogens with different (multiplex) real-time PCR (qPCR) protocols, based on various target genes, as described: *B. burgdorferi* s.l. [24], *B. miyamotoi* [13], *N. mikurensis* [25], *A. phagocytophilum* [26, 27], *Babesia microti* [28], *S. ixodetis* [28], *R. helvetica* [29], and *Babesia* spp. from clade X, which has been designed to detect *Ba. divergens*, *Ba. venatorum* (formerly called EU1-3), *Ba. capreoli* and *Ba. odocoilei* [30]. Samples positive for *B. burgdorferi* s.l. were subjected to conventional PCR and Sanger sequencing of the intergenic spacer region for genospecies identification [31]. Samples positive for *A. phagocytophilum* were subjected to conventional PCR and Sanger sequencing of a fragment of the *GroEL* region for ecotyping [32]. All qPCRs were carried out on a LightCycler 480 (Roche Diagnostics Nederland B.V., Almere, the Netherlands) in a final volume of 20 μl with iQ Multiplex Powermix, 3 μl of sample, and 0.2 μM for all primers and different concentrations for probes [33]. Positive controls and negative water controls were used on every plate tested. To minimise contamination and false-positive samples, the DNA extraction, PCR mix preparation, sample addition, and qPCR analyses were performed in separate air-locked dedicated labs.

We could not test for the presence of tick-borne viruses such as TBE virus and Louping ill virus, as this would have required a more expensive and labour-intensive sampling strategy and testing procedure.

### Comparison with other regions

We sourced data from different studies to compare the presence and prevalence of the tested pathogens in questing *I. ricinus* from Wester Ross, Northwest Scotland, with those in other regions of Scotland, England and Wales, and the Netherlands [28] as an example from mainland Europe. A previously unpublished dataset from 26 sites in Scotland (Grampian and Inverness-shire) was also used for prevalence of *B. burgdorferi* s.l., *A. phagocytophilum*, and *Babesia* species from clade X (Gilbert and Rocchi, unpublished data). For additional *B. burgdorferi* prevalence in Scotland and for the prevalence of all the pathogens in England and Wales, data from published studies were used [21, 25, 34–43]. The ticks from the Netherlands and the ticks from Wester Ross were analysed with the same molecular methods in the same laboratory, following the methods described above, allowing direct comparison of the prevalence. Note that the prevalence from the published literature [21, 25, 34–37, 40–43] and unpublished data from Gilbert and Rocchi were derived using slightly different DNA extraction and PCR protocols, which may have slightly different specificities and sensitivities, so direct comparisons are undertaken with that caveat in mind. Gilbert and Rocchi (unpublished) used the following techniques: DNA from nymphs (pools of three) was extracted using a KingFisher Flex magnetic particle processor (Thermo Scientific) and a MagMAX CORE nucleic acid purification kit (Thermo Fisher). *Anaplasma phagocytophilum* and *B. burgdorferi* s.l. nucleic acids were subjected to specific TaqMan RT-PCR [26], which is the same method used for *A. phagocytophilum* [26] but different from *B. burgdorferi* s.l. in our Wester Ross ticks [24]. *Babesia* spp. DNA was amplified according to Hilpershauser et al. (2020) [44]. Positive samples were Sanger-sequenced (Eurofins MWG) and analysed using DNAStar Lasergene (version 15), and sequences were compared against the BLAST repository (https://blast.ncbi.nlm.nih.gov/Blast.cgi) to obtain parasite speciation. Prevalence estimates of nymphs assumed that a positive pool resulted from one positive nymph, i.e. the number of positive pools divided by the number of nymphs tested for each site. The overall prevalence for each pathogen cited in the results from the Gilbert and Rocchi data was the mean of all the site-level prevalence values.

## Results

We detected the following pathogens tested in the 2828 questing *I. ricinus* ticks from Wester Ross, from highest to lowest prevalence: *A. phagocytophilum* (4.7% prevalence; 132/2828), B*. burgdorferi* s.l. (2.2%; 63/2828), *S. ixodetis* (0.4%; 12/2828)*, Babesia* spp. from clade X (0.2%; 5/2828), and *R. helvetica* (0.04%; 1/2828). See Additional file 1 for more details. Of the *Anaplasma*-positive ticks, 86% (80/93) were identified as the zoonotic ecotype I and 14% (13/93) as the non-zoonotic ecotype II. Four genospecies of *B. burgdorferi* s.l. were detected in the following proportions of positive nymphs: *B. afzelii* (53%, 24/63), *B. valaisiana* (31%, 14/63), *B. garinii* (9%, 4/63), and *B. burgdorferi* s.s. (7%, 3/63). Two genospecies of *Babesia* from clade X were detected, in the following proportions of positive nymphs: *Ba. venatorum* (40%, 2/5) and *Ba. divergens* (60%, 3/5).

### Comparison with other regions

The presence and prevalence of the tick-borne pathogens from other regions of Scotland, England and Wales, and the Netherlands are summarised in Table 1.

**Table 1:** Prevalence (Prev) ± 95% confidence intervals (CI) of the tested pathogens and their respective genospecies in questing *Ixodes ricinus* ticks from Wester Ross (Northwest Scotland) and from other regions of Scotland, England, Wales, and the Netherlands. Presence (+) or absence (−) of each genospecies is indicated if detected in at least one study in each region. Wester Ross (Northwest Scotland) – new data; Other regions of Scotland – combination of published and new data; England and Wales – published data; the Netherlands – published data

Pathogen	Wester Ross (Northwest Scotland)		Other regions of Scotland			England and Wales			The Netherlands		
	Prev	95% CI	Prev	95% CI	References	Prev	95% CI	References	Prev	95% CI	References
*Anaplasma phagocytophilum*	4.7	3.9–5.5	0.7	0.0–1.6	Gilbert and Rocchi (unpubl.) (Grampian and Inverness-shire)^a^	2.3 0.68	1.4–3.5	Hansford et al.2015 [36] (South England) Bown et al. 2008 [41] (Kielder)	5.5	5–6	Krawczyk et al. 2020 [28]
*Ecotype I*	+		NA			NA			+		
*Ecotype II*	+		NA			NA			+		
*Ecotype IV*	−		NA			NA			+		
*Borrelia burgdorferi* s.l.	2.2	1.7–2.8	5.6	NA	James et al.2013 [35] (mainly Grampian and Inverness-shire)	3.9 3.3	2.7–5.5 NA	Hansford et al.2015 [36] (South England) Bettridge et al.2013 [38]	13.3	12.6–14.1	Krawczyk et al. 2020 [28]
			5.9	2.9–9.0	Gilbert and Rocchi (unpubl.) (Grampian and Inverness-shire)^a^	18.1	NA	(North England) Hansford et al.2017 [37] (Salisbury)			
			2.2	1.9–2.6	Gandy 2020 [22] (Grampian)	3.75	NA	Hall 2018 [39] (Lake District overall)			
			1.7	1.4–2.2	Millins et al. 2016 [34] (mainly West and Central)	3.95	3.37–4.59	Cull et al.2021 [40] (England and Wales)			
*B. afzelii*	+		+			+			+		
*B. garinii*	+		+			+			+		
*B. burgdorferi* s.s.	+		+			+			+		
*B. valaisiana*	+		+			+			+		
*Babesia* clade X	0.2	0.06–0.4	0.6	0.0–1.9	Gilbert and Rocchi (unpubl.) (Grampian and Inverness-shire)^a^	0	0–0.36	Bown et al.2008 [41] (Kielder)	0.9	0.7–1.1	Krawczyk et al. 2020 [28]
*Ba. capreoli*	−		NA			NA			+		
*Ba. venatorum*	+		NA			NA			+		
*Ba. odocoilei*	–		NA			NA			+		
*Ba. divergens*	+		NA			NA			+		
*Rickettsia helvetica*	0.04	0–0.2	NA	NA	NA	3	0.95–8.5	Tijsse-Klasen et al.2011 [42] (all England and Scotland)^b^	4.2	3.8–4.7	Krawczyk et al. 2020 [28]
*Spiroplasma ixodetis*	0.4	0.2–0.7	NA	NA	NA	NA	NA	NA	22.9	22–23.9	Krawczyk et al. 2020 [28]
*Neoehrlichia mikurensis*	0.0	< 0.1	NA	NA	NA	0	0–0.4	Hansford et al.2015 [36] (South England)	12.2	11.5–12.9	Krawczyk et al. 2020 [28]
						0	0–3.13	Jahfari et al.2012 [25] (all England and Scotland)			
*Borrelia miyamotoi*	0.0	< 0.1	NA	NA	NA	0.4	NA	Hansford et al.2015 [36] (South England)	3	2.6–3.4	Krawczyk et al. 2020 [28]
						0.6	NA	Hansford et al.2017 [37] (Salisbury)			
						0.2	0.08–0.38	Cull et al.2021[40] (England and Wales)			
*Babesia microti*	0.0	< 0.1	NA	NA	NA	0	0–0.36	Bown et al.2008 [41] (Kielder)	5.1	4.6–5.6	Krawczyk et al. 2020 (28)

Sample sizes as follows: *Anaplasma phagocytophilum*, 18/2142 positive nymphs from 26 sites; *Borrelia burgdorferi* s.l., 238/2264 from 26 sites*; Babesia* clade X, 2/362 from 22 sites

^a^Prevalence values from Gilbert and Rocchi (unpublished) are from nymphs (pools of three), defined as the number of positive pools divided by the number of nymphs tested per site, then averaged over all sites

^b^Although this study was partly carried out in Scotland, all *Rickettsia helvetica*-positive ticks were found in Devon, England

*Anaplasma phagocytophilum* was present at a higher prevalence than in studies from England, and at a similar prevalence as the Netherlands. We found *B. burgdorferi* s.l. prevalence to be lower than in England or the Netherlands, and similar to some other Scottish studies. We found a higher prevalence of *B. valaisiana* and lower prevalence of *B. garinii* than in other studies from Scotland (not shown in table). We found *S. ixodetis* at much lower prevalence than in the Netherlands, and *R. helvetica* at much lower prevalence than in England and the Netherlands.

## Discussion

We revealed the presence of the following tick-borne pathogens in ticks in Wester Ross, Northwest Scotland: *A. phagocytophilum*, *B. burgdorferi* s.l., *Babesia* species from clade X (*Ba. venatorum* and *Ba. divergens*), *R. helvetica*, and *S. ixodetis*, the last being the first record described for Great Britain. This study assessed only the presence of the DNA from the chosen pathogens, and not their viability or infectivity. However, numerous studies already implicate *I. ricinus* as their vector. We cannot assume infectiousness from PCR results, and therefore we cannot translate prevalence estimates of the pathogens to infection risk in humans.

### *Anaplasma phagocytophilum*

The most frequently detected pathogen group was *A. phagocytophilum*, with a prevalence higher than in England and Wales, but comparable to locations in the Netherlands and elsewhere in Europe [45]. The two ecotypes, I and II, are known to circulate in *I. ricinus* [27], and we found both in Wester Ross. The ecotype II is probably non-zoonotic, and most likely maintained by roe deer (*Capreolus capreolus*), which are present at our survey sites in Wester Ross [46]. *Anaplasma phagocytophilum* ecotype I is probably zoonotic and can also cause disease in livestock. It is probably maintained in enzootic cycles by deer species other than roe deer [32]. In Northwest Scotland, red deer (*Cervus elaphus*) are the most common deer species, and likely to be the main natural reservoir host.

### *Borrelia burgdorferi* s.l.

The second most abundant group of tick-borne pathogens was *B. burgdorferi* s.l., with a prevalence of 2.2%. This prevalence is comparable to that reported in two of the four other large cross-sectional surveys from further south and east in Scotland, in the Grampian region (2.2%) [21], and across Scotland but focussing mainly in West and Central Scotland (1.7%) [34]. The other two large-scale Scottish surveys found higher prevalence: 5.6% from across Scotland, but mainly focussed on the Grampian region [35], and 5.9% from Grampian, Moray, and Inverness-shire (Gilbert and Rocchi, unpubl.). Studies from England and Wales showed similar prevalence to studies in Scotland, except in a study from Salisbury (South England), where the prevalence was 18.1% [37]. This higher figure is more comparable to the prevalence from the Netherlands (13.3%) and other parts of continental Western Europe (10.2%) [47].

Similarly to other studies in Scotland [21, 34, 43], the rodent-associated *B. afzelii* was the genospecies that constituted most (53%) of the *B. burgdorferi* s.l. complex of spirochetes in Wester Ross.

We found that in Wester Ross, 7% of the *B. burgdorferi* s.l*.* complex was comprised of *B. burgdorferi* s.s., which is a similar proportion as that reported in all the large Scottish surveys [21, 34, 43]. The reservoir host of *B. burgdorferi* s.s. zoonotic genospecies in Europe has not been firmly established, but recent studies, including from Scotland, suggest a dominant role for squirrels [48–50].

Interestingly, the two bird-associated genospecies, *B. garinii* and *B. valaisiana*, comprised markedly different proportions in Wester Ross from those in previous Scottish surveys, which showed far greater proportions of *B. garinii* (18.2% [21], 28.8% [34], and 36% [43]) and smaller proportions of *B. valaisiana* (7.6% [34], 8% [43], and 10.7% [21]). We found the opposite trends, with only 9% of *B. burgdorferi* s.l. comprising *B. garinii* and 31% comprising *B. valaisiana*. The habitats surveyed in Wester Ross (pine and birch woodlands and open moorland) are similar to those in the other Scottish surveys, and are expected to comprise similar bird species, so we do not know why *B. valaisiana* dominates in Wester Ross as compared to the apparent dominance of *B. garinii* in the rest of Scotland. One possible explanation is competition between the pathogens within the host or the vector, and/or that there is a certain amount of host specificity of each of the bird genospecies between bird species. There is some evidence that *B. garinii* can associate with squirrels [49], so perhaps there are higher squirrel densities in some of the more southern Scottish survey sites than in Wester Ross [51].

The genospecies composition is relevant from a public health perspective, as different genospecies lead to different clinical symptoms in human Lyme disease patients. *Borrelia afzelii* is associated with a skin condition, acrodermatitis chronica atrophicans (ACA), *B. garinii* with neuroborreliosis, and *B. burgdorferi* s.s. with Lyme arthritis [2, 52, 53], whereas *B. valaisiana* may not be pathogenic [54]. As we found a high percentage of *B. afzelii*, general practitioners need to be aware of ACA and its manifestations.

The genospecies composition is also interesting from an ecological perspective. Habitat is an important determinant of vertebrate community composition. All the *B. burgdorferi* s.l.-positive ticks in this study were from mature birch and mature Scots pine woodlands, and both these habitat types are likely to support diverse vertebrate communities, but may differ in their species composition. The low bird-associated *B. garinii* prevalence suggests that rodents play a more important role in Lyme disease hazard than do birds in the study area. Further research on bird and rodent densities at the sites could elucidate the mechanisms driving the relative composition of *Borrelia burgdorferi* s.l. genospecies in Wester Ross.

### *Babesia* species

*Babesia* species from clade X were detected in Wester Ross at a prevalence of 0.2%, compared to 0.6% in other regions of Scotland (Gilbert and Rocchi, unpubl.). Two of the four common *Babesia* species from *I. ricinus* were detected. The first, *Ba. venatorum*, has been associated with some disease cases in humans in Europe [55, 56] and has recently been found in sheep [57] and dogs [58] in the UK*,* although the main natural vertebrate host for *Ba. venatorum* is probably roe deer in Europe [59, 60]. *Babesia divergens,* the second detected genospecies, is of zoonotic importance: it has several zoonotic reservoirs, including deer and cattle, both of which have high cultural and economic value in Wester Ross. It has been found all across Europe affecting cattle [61, 62] as well as humans [61, 63–65], Recently, in July 2020, the first human case of *Ba. divergens* in the UK was reported in Southwest England [66]. *Babesia capreoli* and *Ba. odocoilei* were not detected, but they are present in the Netherlands. No human cases have been associated with either of the two latter pathogens in Europe [67].

### *Rickettsia helvetica*

We found very low prevalence (0.04%) of *R. helvetica* in Wester Ross, Northwest Scotland. This pathogen has previously been detected in ticks in England at a 3% prevalence [42] and in several European countries at prevalence between 4.2% and 16.7%, including the Netherlands (this study), Poland [69], Germany [70], Austria [71], Slovenia [72], and Spain [73], and has been the subject of several cases in humans with febrile illness and perimyocarditis [74–76]. It is therefore considered an emerging human pathogen [77]. As *R. helvetica* is efficiently transmitted vertically in ticks (i.e. transovarial and trans-stadial transmission), ticks themselves can be considered as reservoirs hosts [68]. Which vertebrate hosts contribute most to the life cycle of *R. helvetica* has not be fully investigated yet, but the pathogen has been detected in bank vole, wood mouse, shrew, wild boar, and roe deer [68, 78, 79]. The prevalence of *R. helvetica* is very low, and one may wonder whether or how a tick-borne pathogen can be maintained in an enzootic cycle with such a low prevalence. One possible explanation is that ticks with *R. helvetica* are not maintained in a regional enzootic cycle but (continuously) introduced by migratory birds [80].

### *Spiroplasma ixodetis*

We detected the relatively unknown tick-borne pathogen *S. ixodetis* (0.4% prevalence), and this is the first record for Great Britain. The bacterial parasite *Spiroplasma ixodetis* was first isolated from *Ixodes pacificus* ticks in the USA in 1995 [81], and it has previously been found in Slovakia in all active life stages of *I. ricinus* including larvae [82–84]*,* which suggests that transmission can be transovarial. The role of hosts in the *S. ixodetis* transmission cycle is unknown [28]. Several European studies have described human cases of cataract and uveitis in newborn babies that showed evidence of transplacental infection with *S. ixodetis* [85, 86].

### *Neoehrlichia mikurensis*, *Borrelia miyamotoi*, and *Babesia microti*

Although we did not observe *N. mikurensis*, *B. miyamotoi*, or *Ba. microti* in questing nymphs from Wester Ross, this cannot necessarily be interpreted as a complete absence of enzootic circulation of these pathogens, because the prevalence of some of these pathogens can be low, for example, 0.1% for *N. mikurensis* in regions of Denmark [87], 0.6% for *Ba. microti* in Poland [88] and 1.5% in the UK (ticks pulled from dogs) [89], and 0.8% for *B. miyamotoi* in regions of Germany [90] and 0.5% in England (Table 1). In our sample of 2828 ticks, a prevalence of 0.1% would mean that there would be an expectation of around three ticks that test positive in our sample size. Therefore, it could be that the pathogens are present but were undetected, especially if the circulation of the pathogens is very localised or occurs in habitats insufficiently monitored in this study. In a separate study, also in Wester Ross, we did find *Ba. microti* in one engorged tick pulled from a wood mouse (*Apodemus sylvaticus*) (Olsthoorn et al. unpubl.). *Neoehrlichia mikurensis* has so far not been found in any study that tested for it in Great Britain [36, 37], so there is a realistic possibility that this pathogen is not circulating in Scotland.

We tested 8282 questing nymph and adult ticks, and it is possible that a tiny proportion of the ticks analysed may have been non-*ricinus*, although this is highly unlikely, as previous studies from Scotland identified 100% of questing ticks (9883 in total) to be *I. ricinus* [20–22]*.*

## Conclusions

In the first surveys of tick-borne pathogens in Northwest Scotland, in a region much frequented by tourists, we identified and quantified the prevalence of *A. phagocytophilum*, *B. burgdorferi* s.l.*, Babesia* species from clade X (*Ba. venatorum* and *Ba. divergens*), *R. helvetica*, and *S. ixodetis*, the latter being the first record described for Great Britain. *Anaplasma phagocytophilum* occurred at higher prevalence than elsewhere in Great Britain, while *B. burgdorferi* s.l. was less prevalent than in England or the Netherlands, though similar to other Scottish studies. This new knowledge has implications for public and veterinary health in terms of improving diagnoses and treatment and better targeting of awareness and mitigation strategies.

Further research is needed to investigate the life cycles and mechanisms of transmission of these pathogens. *Anaplasma phagocytophilum* and *Ba. divergens*, both of zoonotic importance, are transmitted by deer, an important cultural and economic asset in the Wester Ross area as well as other parts of Europe. *Rickettsia helvetica* has been found in several vertebrates, but its life cycle is unclear and needs further investigation. More studies in different habitats are needed to shed light on the ecological determinants of genospecies composition of *B. burgdorferi* s.l.

## Supplementary Information

**Additional file 1:Text S1.** Field methods explained. **Table S1.** Sum of ticks collected at each visit on each plot is summarised, as well as the sum of ticks positive for each of the eight pathogens tested for in the study, namely *Borrelia miyamotoi*, *Borrelia burgdorferi* s.l., *Anaplasma phagocytophilum*, *Neoehrlichia mikurensis*, *Babesia* spp. from clade X, *Spiroplasma ixodetis*, *Babesia microti*, and *Rickettsia helvetica*. **Table S2.** Molecular analysis results of the 2828 adult and nymph ticks.

## Data Availability

The datasets used and/or analysed during the current study are available from the corresponding author on reasonable request.
