# Supplementary material for: Occurrence of tick-borne pathogens in questing Ixodes ricinus ticks from Wester Ross, Northwest Scotland
Source: Parasit Vectors. 2021 Aug 26;14:430. doi: 10.1186/s13071-021-04946-5 (PMC8393815; doi:10.1186/s13071-021-04946-5)
Supplement: Supplementary file 1 — Additional file 1:Text S1. Field methods explained. Table S1. Sum of ticks collected at each visit on each plot is summarised, as well as the sum of ticks positive for each of the eight pathogens tested for in the study, namely Borrelia miyamotoi, Borrelia burgdorferi s.l., Anaplasma phagocytophilum, Neoehrlichia mikurensis, Babesia spp. from clade X, Spiroplasma ixodetis, Babesia microti, and Rickettsia helvetica. Table S2. Molecular analysis results of the 2828 adult and nymph ticks. [file 13071_2021_4946_MOESM1_ESM.docx]

**Additional file 1: Field methods and results of molecular analysis of the 8 pathogen groups**

Text S1: We conducted surveys of ticks on 26 sites in Wester Ross, Northwest Scotland (a subset of sites from a larger study), consisting of the following four land cover types: open land dominated by different heather species (*Erica* spp. and *Calluna* spp.) (8 sites) , young Scots pine (*Pinus sylvestris*) between 13 and 30 years old (2 sites), mature Scots pine woodland of minimum 60 years old (8 sites) and mature deciduous birch woodland (*Betula* spp.) (8 sites). We visited the plots in 2018 and/or 2019 in late Spring (May), early Summer (June/July) and/or late Summer (August). Table S1 shows details on tick collection sites and Table S2 shows details on molecular analysis results.

Table S1: Sum of ticks collected at each visit on each plot is summarised, as well as the sum of ticks positive for each of the eight pathogens tested for in the study, namely *Borrelia miyamotoi, Borrelia burgdorferi* s.l., *Anaplasma phagocytophilum, Neoehrlichia mikurensis, Babesia* spp from clade X, *Spiroplasma ixodetis, Babesia microti* and *Rickettsia helvetica*.

| **Year** | **Sampling session** | **Site code** | **Site type** | **Sum of ticks from site (adults and nymphs)** | ***Borrelia miyamotoi* positives** | ***Borrelia burgdorferi* s.l. positives** | ***Anaplasma phagocytophilum* positive** | ***Neoehrlichia mikurensis* positives** | ***Babesia* from Clade X positives** | ***Spiroplasma ixodetis* positives** | ***Babesia microti* positives** | ***Rickettsia helvetica* positives** |
| --- | --- | --- | --- | --- | --- | --- | --- | --- | --- | --- | --- | --- |
| 2018 | late Spring | BM1 | mature birch | 472 | 0 | 2 | 16 | 0 | 0 | 0 | 0 | 0 |
| 2018 | early Summer | BM1 | mature birch | 369 | 0 | 0 | 22 | 0 | 0 | 0 | 0 | 0 |
| 2018 | mid-Summer | BM1 | mature birch | 112 | 0 | 0 | 23 | 0 | 1 | 0 | 0 | 0 |
| 2018 | late Spring | BM2 | mature birch | 70 | 0 | 1 | 5 | 0 | 0 | 0 | 0 | 0 |
| 2018 | early Summer | BM2 | mature birch | 7 | 0 | 0 | 0 | 0 | 0 | 1 | 0 | 0 |
| 2018 | mid-Summer | BM2 | mature birch | 11 | 0 | 0 | 0 | 0 | 0 | 0 | 0 | 0 |
| 2018 | late Spring | BM3 | mature birch | 179 | 0 | 8 | 1 | 0 | 0 | 0 | 0 | 0 |
| 2018 | early Summer | BM3 | mature birch | 85 | 0 | 7 | 1 | 0 | 1 | 0 | 0 | 0 |
| 2018 | mid-Summer | BM3 | mature birch | 71 | 0 | 4 | 6 | 0 | 0 | 0 | 0 | 0 |
| 2018 | late Spring | BM4 | mature birch | 80 | 0 | 4 | 1 | 0 | 0 | 3 | 0 | 0 |
| 2018 | early Summer | BM4 | mature birch | 86 | 0 | 5 | 1 | 0 | 0 | 3 | 0 | 0 |
| 2018 | mid-Summer | BM4 | mature birch | 42 | 0 | 9 | 0 | 0 | 0 | 0 | 0 | 0 |
| 2018 | late Spring | BM5 | mature birch | 9 | 0 | 0 | 0 | 0 | 0 | 0 | 0 | 0 |
| 2018 | early Summer | BM5 | mature birch | 8 | 0 | 0 | 0 | 0 | 0 | 0 | 0 | 0 |
| 2018 | mid-Summer | BM5 | mature birch | 11 | 0 | 1 | 0 | 0 | 0 | 0 | 0 | 0 |
| 2018 | late Spring | BM6 | mature birch | 52 | 0 | 0 | 0 | 0 | 0 | 0 | 0 | 0 |
| 2018 | early Summer | BM6 | mature birch | 80 | 0 | 1 | 1 | 0 | 0 | 0 | 0 | 0 |
| 2018 | mid-Summer | BM6 | mature birch | 59 | 0 | 1 | 2 | 0 | 0 | 0 | 0 | 0 |
| 2019 | mid-Summer | BM6 | mature Scots pine | 80 | 0 | 0 | 2 | 0 | 1 | 0 | 0 | 0 |
| 2018 | early Summer | BM7 | mature birch | 13 | 0 | 0 | 1 | 0 | 0 | 0 | 0 | 0 |
| 2018 | early Summer | BM8 | mature birch | 89 | 0 | 3 | 1 | 0 | 0 | 1 | 0 | 0 |
| 2019 | mid-Summer | BM8 | mature Scots pine | 11 | 0 | 2 | 1 | 0 | 0 | 0 | 0 | 0 |
| 2018 | early Summer | H1 | open land | 3 | 0 | 0 | 0 | 0 | 0 | 0 | 0 | 0 |
| 2018 | early Summer | H2 | open land | 10 | 0 | 0 | 0 | 0 | 0 | 0 | 0 | 0 |
| 2018 | early Summer | H3 | open land | 5 | 0 | 0 | 0 | 0 | 0 | 0 | 0 | 0 |
| 2018 | mid-Summer | H4 | open land | 12 | 0 | 0 | 0 | 0 | 0 | 0 | 0 | 0 |
| 2018 | early Summer | H5 | open land | 43 | 0 | 2 | 5 | 0 | 0 | 1 | 0 | 0 |
| 2018 | early Summer | H6 | open land | 2 | 0 | 0 | 0 | 0 | 0 | 0 | 0 | 0 |
| 2018 | early Summer | H7 | open land | 10 | 0 | 0 | 0 | 0 | 0 | 0 | 0 | 0 |
| 2018 | early Summer | H8 | open land | 1 | 0 | 0 | 0 | 0 | 0 | 0 | 0 | 0 |
| 2018 | early Summer | PM1 | mature Scots pine | 6 | 0 | 0 | 0 | 0 | 0 | 0 | 0 | 0 |
| 2018 | early Summer | PM2 | mature Scots pine | 13 | 0 | 0 | 0 | 0 | 0 | 0 | 0 | 0 |
| 2018 | early Summer | PM3 | mature Scots pine | 125 | 0 | 4 | 12 | 0 | 1 | 0 | 0 | 0 |
| 2018 | mid-Summer | PM3 | mature Scots pine | 31 | 0 | 1 | 4 | 0 | 0 | 0 | 0 | 0 |
| 2019 | mid-Summer | PM3 | mature Scots pine | 110 | 0 | 3 | 6 | 0 | 1 | 1 | 0 | 0 |
| 2018 | mid-Summer | PM4 | mature Scots pine | 37 | 0 | 0 | 1 | 0 | 0 | 0 | 0 | 0 |
| 2019 | mid-Summer | PM4 | mature Scots pine | 23 | 0 | 1 | 0 | 0 | 0 | 0 | 0 | 0 |
| 2018 | early Summer | PM5 | mature Scots pine | 14 | 0 | 0 | 0 | 0 | 0 | 1 | 0 | 0 |
| 2018 | early Summer | PM6 | mature Scots pine | 52 | 0 | 0 | 8 | 0 | 0 | 1 | 0 | 0 |
| 2018 | mid-Summer | PM6 | mature Scots pine | 71 | 0 | 0 | 2 | 0 | 0 | 0 | 0 | 1 |
| 2018 | early Summer | PM7 | mature Scots pine | 51 | 0 | 0 | 2 | 0 | 0 | 0 | 0 | 0 |
| 2018 | mid-Summer | PM7 | mature Scots pine | 69 | 0 | 0 | 2 | 0 | 0 | 0 | 0 | 0 |
| 2019 | mid-Summer | PM7 | mature Scots pine | 40 | 0 | 4 | 0 | 0 | 0 | 0 | 0 | 0 |
| 2018 | early Summer | PM8 | mature Scots pine | 81 | 0 | 0 | 5 | 0 | 0 | 0 | 0 | 0 |
| 2018 | mid-Summer | PM8 | mature Scots pine | 2 | 0 | 0 | 0 | 0 | 0 | 0 | 0 | 0 |
| 2018 | early Summer | PY1 | young Scots pine | 15 | 0 | 0 | 1 | 0 | 0 | 0 | 0 | 0 |
| 2018 | early Summer | PY2 | young Scots pine | 6 | 0 | 0 | 0 | 0 | 0 | 0 | 0 | 0 |

Table S2: Molecular analysis results of the 2828 adult and nymph ticks

| **Pathogen** | **Total ticks tested** | **Total positives** | **% positives** | **Lower CI 2.5%** | **Upper CI 97.5%** |
| --- | --- | --- | --- | --- | --- |
| ***Borrelia burgdorferi* s.l.** | **2828** | **63** | **2.23** | **1.72** | **2.84** |
| *B. afzelii* | 45 out of 63 positives | 24 | 53.30 | 37.87 | 68.34 |
| *B. garinii* | 45 out of 63 positives | 4 | 8.89 | 2.48 | 21.22 |
| *B. burgdorferi* s.s. | 45 out of 63 positives | 3 | 6.67 | 1.4 | 18.27 |
| *B.valaisiana* | 45 out of 63 positives | 14 | 31.11 | 18.17 | 46.65 |
| ***Borrelia miyamotoi*** | **2828** | **0** | **0.00** | **0** | **0.13** |
| ***Anaplasma phagocytophilum*** | **2828** | **132** | **4.67** | **3.92** | **5.51** |
| *Anaplasma* ecotype 1 | 93 out of 132 positives | 80 | 86.02 | 77.28 | 93.34 |
| *Anaplasma* ecotype 2 | 93 out of 132 positives | 13 | 13.98 | 7.66 | 22.72 |
| *Anaplasma* ecotype 4 | 93 out of 132 positives | 0 | 0.00 | 0 | 0.13 |
| ***Neoehrlichia mikurensis*** | **2828** | **0** | **0** | **0** | **0.13** |
| ***Babesia from clade X*** | **2828** | **5** | **0.18** | **0.06** | **0.41** |
| *Ba. capreoli* | 5 out of 5 positives | 0 | 0 | 0 | 0.13 |
| *Ba. venatorum* | 5 out of 5 positives | 2 | 40 | 5.27 | 85.34 |
| *Ba. odocoilei* | 5 out of 5 positives | 0 | 0 | 0 | 0.13 |
| *Ba. divergens* | 5 out of 5 positives | 3 | 60 | 14.66 | 94.73 |
| ***Spiroplasma Ixodetis*** | **2828** | **12** | **0.42** | **0.22** | **0.74** |
| ***Babesia microti*** | **2828** | **0** | **0** | **0** | **0.13** |
| ***Rickettsia helvetica*** | **2828** | **1** | **0.04** | **0** | **0.02** |
